# Effect of Harvey Rat Sarcoma Virus Mutation in Oral Squamous Cell Carcinoma and Its Influence on Different Populations: A Systematic Review

**DOI:** 10.7759/cureus.45505

**Published:** 2023-09-18

**Authors:** Mudiyayirakkani Muthusamy, Pratibha Ramani, Paramasivam Arumugam

**Affiliations:** 1 Department of Oral and Maxillofacial Pathology, Saveetha Dental College and Hospitals, Chennai, IND; 2 Department of Oral Pathology and Microbiology, Saveetha Dental College and Hospitals, Saveetha Institute of Medical and Technical Sciences, Saveetha University, Chennai, IND; 3 Centre for Cellular and Molecular Research, Saveetha Dental College and Hospitals, Chennai, IND

**Keywords:** gtp-ase activating protein, signaling pathway, gene mutation, oral squamous cell carcinoma, hras

## Abstract

The information for protein synthesis is given by the genes. These proteins are responsible for controlling functions like cell growth, differentiation, cell maturation, and cell death. In the case of genetic mutations, the protein functions get disturbed leading to a drastic shift in the normal physiological functions of cell growth, differentiation, and proliferation, making the normal cell cancerous. The Harvey rat sarcoma virus (HRAS) gene is an oncogene that belongs to the rat sarcoma virus (RAS) family. HRAS gene provides the instructions for making the HRAS protein that plays an important role in regulating cell division and when the HRAS gene gets mutated it gets involved in initiating cancer. HRAS mutation has been frequently noted in head and neck cancers; however, the mechanism of HRAS mutation involved in the initiation of oral squamous cell carcinoma (OSCC) still remains unexplored. An elaborate systematic literature search was done in PubMed, Scopus, and Web of Science databases. It was found that the Ras-dependent mutations affect the involved upstream and downstream components of the Ras-Raf-MAPK (rat sarcoma virus-rapidly accelerated fibrosarcoma-mitogen-activated protein kinase) pathway deregulating the signal leading to tumorigenesis. The Ras mutation can affect the Ras-Raf-MAPK pathway at different stages. The disease caused is based on the frequency of the HRAS mutation and it can lead to diverse cellular outcomes as it is mainly associated with cell division, differentiation, growth, survival, and the cell cycle. The crosstalk between the signaling pathways is controlled by the signaling molecules resulting in the creation of molecular networks. The balance of these molecular networks is very important to determine the cellular outcome. This systematic review inspects the molecular network of HRAS and its vital role in carcinogenesis. It is aimed at exploring and summarizing the contributions of the HRAS mutation involved in the pathogenesis of OSCC.

## Introduction and background

Cancer is one of the leading causes of morbidity and mortality in the world. In the year 2020, a global cancer survey by the Global Cancer Observatory (GLOBOCAN) reported around 19.3 million new cancer cases [1]. The cancer burden is increasing rapidly, and its incidence and mortality are expected to rise to 29.5 million and 16.3 million by the year 2040. Oral cancer ranks 11th in prevalence when compared to other cancers in the world [2]. According to the National Cancer Registry Programme 2022, oral cancer is the second most common cancer in India [3]. It is estimated that around 1,45,844 new oral cancer cases were reported in males and around 52,594 new oral cancer cases were reported in females in 2022 [3]. Oral cancer makes up nearly 40% of all cancers in India, where it is the third most common disease in women and the most common cancer in males. According to Petersen et al., the age-standardized incidence rate of oral cancer in India is 12.6 per 1,00,000 people, and the pace is quickly rising [4]. Oral squamous cell carcinoma (OSCC) makes up more than 90% of all oral cancer cases [5]. It appears on the tongue, alveolar ridges, lips, buccal mucosa, floor of the mouth, retromolar area, hard palate, and soft palate. OSCC is a multifactorial disease that is caused by several risk factors like smoking, alcohol usage, betel nut use, diet and nutrition, radiation exposure, genetic mutations, free radicals, chronic inflammation, oral microflora as well as occupational and dental variables [6]. The pathophysiology of OSCC due to genetic mutations remains an enigma. The two most common classes of genes responsible for the development of tumors are the oncogenes and tumor suppressor genes. The oncogenes code for proteins that drive the cell cycle. In normal physiological processes, the proto-oncogenes play a critical role in proceeding the cell cycle in the forward direction from G (gap) phases to either chromosomal replication (S phase) or chromosome segregation (mitosis). Once the normal physiological process is disturbed due to mutation of the proto-oncogene, it gets changed to an oncogene. The oncogenes code for abnormal protein products that exhibit increased activity contributing to tumor development. One such oncogene is the rat sarcoma virus (RAS) gene.

**Figure 1:**
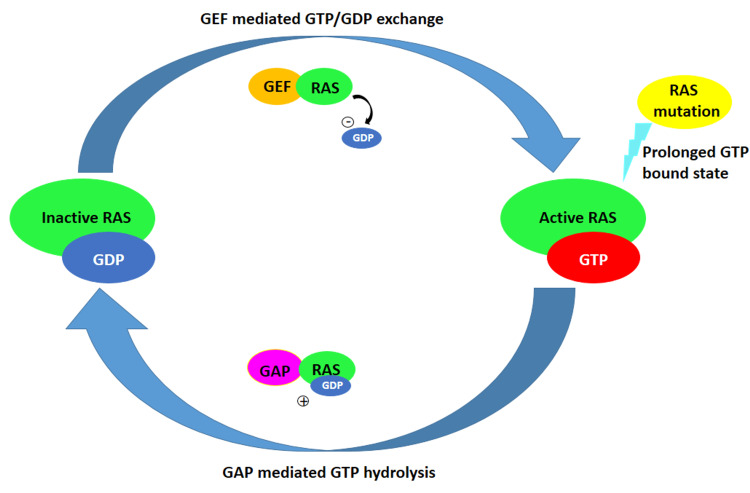
Mechanism of RAS activation The figure shows the mechanism of activation and inactivation of RAS with the help of guanine nucleotide exchange factors (GEFs) and by binding guanosine triphosphate (GTP). The inactivation of RAS is mediated by the GTPase-activating proteins (GAPs) and hydrolysis of GTP to guanosine diphosphate (GDP). Prolonged GTP-bound state (active) of RAS leads to mutation, "-" represents the release of GDP during the GDP/GTP exchange, and "+" represents the hydrolysis of GTP to GDP.

The Ras proteins are located in the inner plasma membrane of the cells. The opposing actions of guanine nucleotide exchange factors (GEFs) and GTPase-activating proteins (GAPs), which encourage the acquisition of the active guanosine triphosphate (GTP) and inactive guanosine diphosphate (GDP)-bound conformations, respectively, control their activity in a cyclic manner based on the response to the inside-out or outside-in signals [7]. The Ras superfamily of small GTPases acts as a binary molecular switch, which is turned on (active Ras) by binding GTP and off (inactive Ras) by hydrolyzing GTP to GDP (Figure 1). An essential function of the Ras superfamily is the post-translational modification by lipids [8]. The Ras proteins are stimulated by external stimuli and interact with the downstream effectors to regulate physiological functions like controlling cellular proliferation, differentiation, survival, and apoptosis through signal transduction. Ras switches between the active (GTP) form and the inactive (GDP) form quickly in healthy cells by receiving signals and acting upon them. When the Ras gene is altered, the Ras remains in the active state (GTP form), preventing cells from responding to death signals and resulting in uncontrolled cell proliferation. After mutation, there is an accelerated intrinsic GTP/GDP exchange, co-activation of wild-type RAS by Son of Sevenless (SOS), GEFs, impaired intrinsic GAP-mediated GTP hydrolytic property, and GAP insensitivity [9].

**Figure 2:**
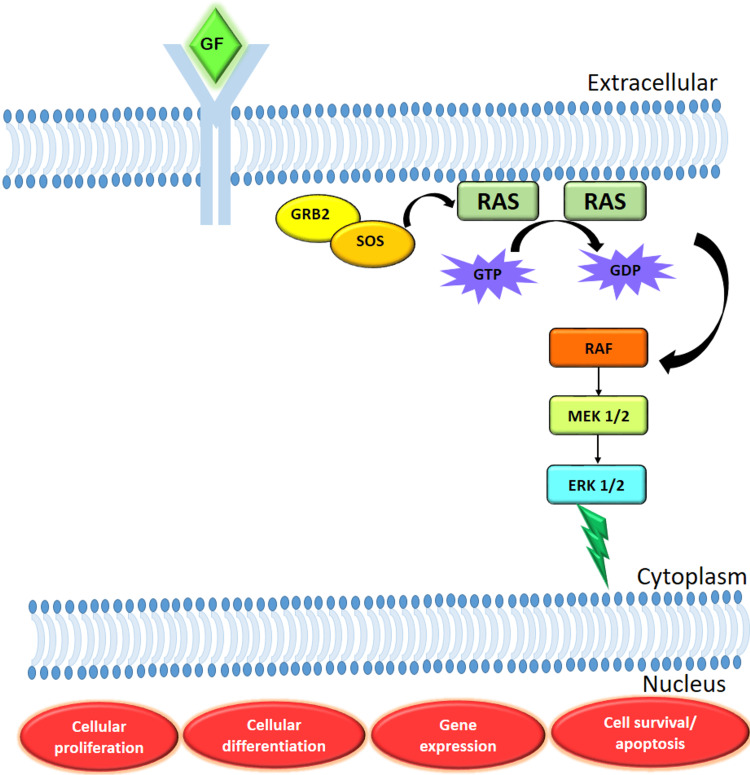
The Ras/Raf/MAPK pathway This figure shows the rat sarcoma virus/rapidly accelerated fibrosarcoma/mitogen-activated protein kinase (Ras/Raf/MAPK) pathway that leads to cellular proliferation, cellular differentiation, gene expression, and cell survival/apoptosis. When the growth factor (GF) binds to the receptor, the growth factor receptor bound protein 2 (GRB2) and Son of sevenless (SOS) activate the rat sarcoma virus (RAS) from its inactive state to active state during which there is a release of GDP. The activated RAS then activates the rapidly accelerated fibrosarcoma (RAF) which accelerates the activation of mitogen-activated protein kinase kinase (MEK) that in turn induces the activation of extracellular signal-regulated kinase (ERK). This activation of ERK makes nuclear changes leading to cellular proliferation, cellular differentiation, gene expression, and cell survival/apoptosis.

Three RAS proteins are expressed in our body. The three Ras genes are the Harvey rat sarcoma virus (HRAS), Kirsten rat sarcoma virus (KRAS), and neuroblastoma ras viral oncogene (NRAS), respectively. The HRAS gene involved in the rat sarcoma virus- rapidly accelerated fibrosarcoma-mitogen-activated protein kinases (Ras-Raf-MAPK) and the phosphoinositide-3 kinase (PI3K) pathways decide the critical life process of cells. These pathways are responsible for the signal transduction. In the case of mutated HRAS, these pathways get deregulated leading to tumorigenesis. Around 30% of all human tumors are associated with a mutation in RAS genes [10]. KRAS mutation is responsible for 32% of lung cancers, 40% of colorectal cancers, and 85%-90% of pancreatic cancers [11-13]. HRAS mutations are comparatively less frequent than KRAS and NRAS mutations but in head and neck cancers, HRAS mutations are most frequently detected [14,15]. The HRAS oncogene produces the proteins necessary for the process of transducing extracellular signals for growth, differentiation, and survival (Figure 2). The small GTPases and the lipid kinase PI(3)K, which are crucial for signal transduction, are also activated by the RAS-activated receptor tyrosine kinase. The phosphatidylinositol-3-kinase (PI3K)/AKT and MAPK pathways are further stimulated by this activated RAS. Phosphorylation of mitogen-activated protein kinase kinase (MEK) 1/2 by RAF1 kinase is the most critical downstream step. It takes place on two different serine residues. This in turn activates the phosphorylation of extracellular signal-regulated kinase (ERK) 1/2 on threonine and tyrosine residues which results in cell growth and differentiation [16]. We aim this systematic review to understand the effect of HRAS mutation in the pathogenesis of OSCC.

## Review

Search strategy

The databases used for the literature search were PubMed, Scopus, and Web of Science. The articles related to this topic were collected from its inception till December 2022. The search terms that were used to obtain the results were oral cancer OR oral squamous cell carcinoma OR oral squamous cell carcinoma of buccal mucosa OR oral squamous cell carcinoma of tongue OR oral squamous cell neoplasm[MeSH Terms] OR oral squamous cell malignancy[MeSH Terms] OR oral carcinoma[MeSH Terms] OR oral squamous cell carcinoma of hard palate[MeSH Terms] OR oral squamous cell carcinoma of soft palate[MeSH Terms] OR oral tumor[MeSH Terms] OR oral malignancy[MeSH Terms] OR oral neoplasm[MeSH Terms] OR alveolar carcinoma[MeSH Terms] OR alveolar cancer[MeSH Terms] OR gingivobuccal carcinoma[MeSH Terms] OR gingivobuccal tumor[MeSH Terms] OR cheek tumor[MeSH Terms] OR cheek cancer[MeSH Terms] OR tongue neoplasm[MeSH Terms] OR tongue tumor[MeSH Terms] OR tongue cancer[MeSH Terms] OR head and neck neoplasm[MeSH Terms] OR head and neck carcinoma[MeSH Terms] OR lingual tumor[MeSH Terms] OR lingual cancer[MeSH Terms] OR lingual carcinoma[MeSH Terms] OR buccal neoplasm[MeSH Terms] OR buccal tumor[MeSH Terms] OR buccal cancer[MeSH Terms] AND HRAS OR C-H-RAS OR Harvey murine sarcoma virus oncogene OR Harvey rat sarcoma viral oncogene homolog OR HRAS1 OR Oncogene, G-RAS OR RASH1 OR RASH_HUMAN OR Transformation gene: Oncogene HaMSV OR Transforming protein P21/H-RAS-1 C-H-RAS OR v-Ha-ras Harvey rat sarcoma viral oncogene homolog, which resulted in 163 articles.

Inclusion and exclusion criteria

*Inclusion Criteria* 

Original research paper publications, observational studies in cross-sectional, case-control, and cohort study designs, reported outcome or one of the outcomes as HRAS mutation in OSCC, and studies reporting histopathologically confirmed OSCC cases.

Exclusion Criteria

Letters, retracted articles, reviews, and animal studies were excluded. Studies with insufficient data and duplicate results were also excluded.

Data extraction

The data were screened and retrieved by two investigators independently. The data required were extracted from each paper by two independent investigators. Information like the name of the first author, year of publication, study design, sample size, number of included cases, demographic characteristics of the included participants (age, gender), and methods used for the analysis of the mutation were also extracted from each included article.

Quality assessment of the included studies (risk of bias)

The Newcastle Ottawa Scale was the tool that was used for quality assessment (risk of bias). In cross-sectional studies, the domains of selection, comparability, and outcome were evaluated for each study. Domains like sample representativeness, sample size, response rate, and determination of the screening or surveillance tool were evaluated under selection. Under outcome, areas including outcome evaluation and statistical testing were evaluated. In case-control studies, selection, comparability, and exposure were among the domains that were evaluated for each study. Assessed under selection were areas including adequate case definition, case representativeness, control selection, and control definition. Domains related to exposure, such as determining exposure, using the same approach to determine exposure for cases and controls and non-response rate, were evaluated. (Each included cross-sectional study's domains are listed in Table 1, and each included case-control study's domains are included in Table 2.)

**Table 1:** Quality assessment of the cross-sectional studies This table shows the quality assessment of the included cross-sectional studies using the Newcastle Ottawa Scale. "*" represents the score for the respective domain. Each "*" has a score value of 1. On adding all the "*" of an article gives the summary score. "*" represents score 1, "**" represents score 2, and "***" represents score 3, respectively.

S. No	Authors of publication	Selection				Comparability	Outcome		Summary scores
		Representativeness of sample	Sample size	Ascertainment of the exposure	Non-respondents	The subjects in different outcome groups are comparable based on the study design or analysis, confounding factors are controlled	Assessment of outcome	Statistical tests	
1.	Chang et al. [17]	*		**		*	**	*	7
2.	Murugan et al. [18]	*	*	**	*	*	**	*	9
3.	Warnakulasuriya et al. [19]	*		**	*	*	**	*	8
4.	Zanaruddin et al. [20]	*	*	**	*	*	**	*	9
5.	Saranath et al. [21]	*		**	*	*	**	*	8
6.	Munirajan et al. [22]	*	*	**	*	*	**	*	9
7.	Sathyan et al. [23]	*	*	**	*	*	**	*	9

**Table 2:** Quality assessment of the case-control studies This table shows the quality assessment of the included case-control studies using the Newcastle Ottawa Scale. "*" represents the score for the respective domain. Each "*" has a score value of 1. Adding all the "*" of an article gives the summary score. "*" represents score 1, "**" represents score 2, and "***" represents score 3, respectively.

S. No	Authors and year of publication	Selection				Comparability	Exposure			Summary scores
		Adequate case definition	Representativeness of the cases	Selection of controls	Definition of controls	Comparability of cases and controls on the basis of the design and analysis	Ascertainment of exposure	The same method of ascertainment for cases and controls	Non-response rate	
1.	Koumaki et al. [24]	*	*	*	*	*	*	*	*	8
2.	Uchibori et al. [25]	*	*	*	*	*	*	*	*	8

Results

Results of the Literature Search

**Figure 3:**
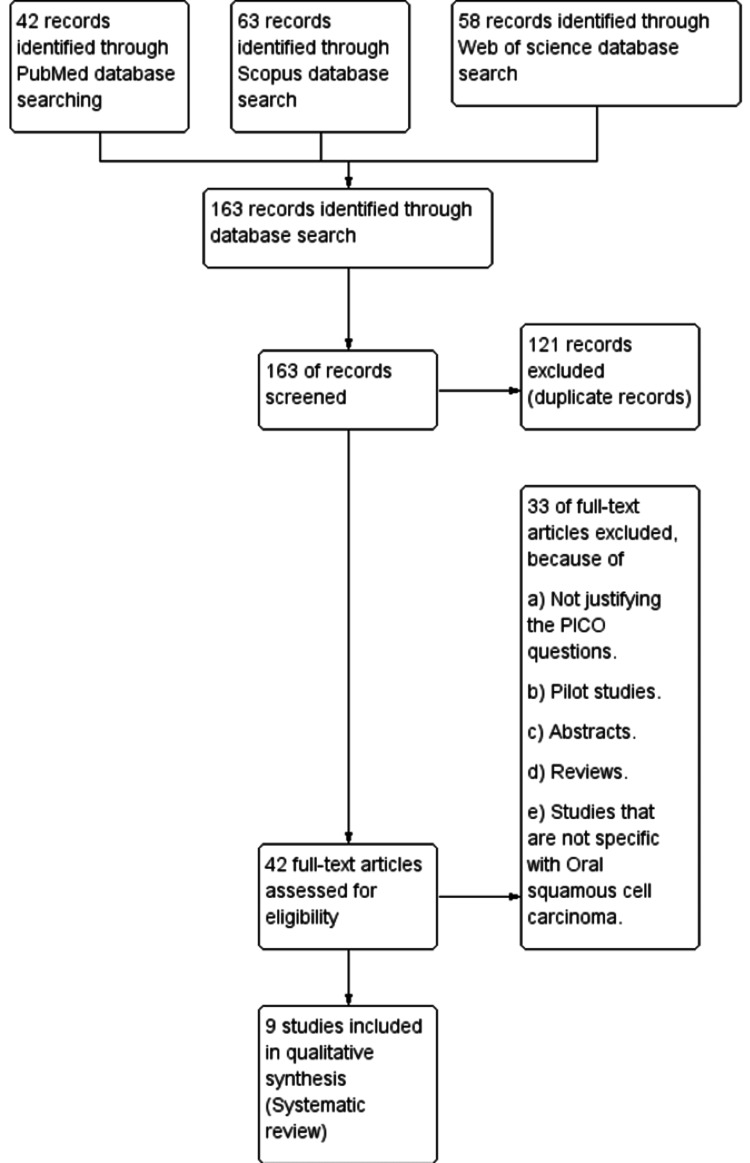
PRISMA flowchart emphasizing the steps in the critical screening of the articles. This figure explains the method of literature search and screening of the articles to be included in the study.

The retrieval of the articles of the included studies of this systematic review is summarized in the PRISMA flow chart (Figure 3). A total of 163 articles were identified, 42 in PubMed, 63 in Scopus, and 58 in Web of Science, respectively. From the 163 records selected for screening, around 121 records were not eligible for the systematic review and were eliminated. From the remaining 42 records, furthermore, 33 full-text records were eliminated because of reasons like not matching the PICO questions, pilot studies, reviews, abstracts, and studies that are not specific to OSCC. Finally, after a critical eligibility assessment and detailed analysis, nine articles were included in this systematic review.

Results of the Quality Assessment of the Included Studies

Quality assessment of the included studies was done by using the Newcastle Ottawa Scale. The scores of the included studies were converted to Agency for Healthcare Research and Quality (AHRQ) standards based on the following thresholds: Good quality: 2 or 3 stars in the outcome/exposure domain, 1 or 2 stars in the comparability domain, 3 or 4 stars in the selection domain; Fair quality: 2 or 3 stars in the outcome/exposure, 1 or 2 stars in the comparability domain, 2 stars in the selection domain; poor quality: 0 or 1 star in the outcome/exposure domain OR 0 star in the comparability domain OR 0 or 1 star in the selection domain. Based on the scores of the included studies, it can be seen that all the included studies are of good quality.

Interpretation

The included studies have a total of 895 participants enrolled (Table 3). Out of the total enrolled participants, 86 (9.6%) participants exhibited HRAS mutation. Among the Taiwanese population, 10 cases showed missense mutation of the HRAS gene in which six mutations were single mutations and two mutations were in combination with the phosphatidylinositol-4,5-bisphosphate 3-kinase catalytic subunit alpha (PIK3CA) mutation. The frequency of HRAS mutation also significantly correlated with the tumor grade (p = 0.030) [17]. Vietnamese population exhibited one insertion mutation, nine point mutations, and 12 silent mutations of the HRAS gene. The association between the disease staging and the HRAS mutation was statistically significant (p = 0.0322) where nine mutations were found in stages III and IV of the disease and one mutation was found in stages 0-II of the disease [18]. The Caucasoid population showed one point mutation and in the Asian population, 3 (2.4%) exhibited HRAS mutations in glycine to serine at amino acid position 12 (G12S) and glycine to aspartic acid at amino acid position 12 (G12D) [19,20]. The Indian population exhibited 20 HRAS mutations, among which eight samples were mutated in codon 12, one sample at codon 13, and 13 samples at codon 61. Two samples showed concurrent mutations in codons 12 and 61 and also in another study on the Indian population exhibited 8 (17.39%) HRAS mutations, 6 (13.04%) of the mutations were seen in codon 12, 1 (2.17%) mutation was seen in codon 13, and 1 (2.17%) mutation was seen in codon 59 [21,22]. In the Arabian population, 19 HRAS mutations were seen and the HRAS mutation was significantly associated with the expression of cell-cycle regulatory proteins like decreased expression of cyclin D1 (p = 0.027) and cyclin-dependent kinase 4 (CDK4) (p = 0.046) and overexpression of retinoblastoma (Rb) (p = 0.011) and p16 (p = 0.026), respectively. It was also found that the HRAS mutant carriers also exhibit significantly high recurrence-free survival (p = 0.033) [23]. The Greek population exhibited 10 silent mutations, two hotspot mutations, and one missense mutation [24]. The Japanese population exhibited one HRAS mutation and the association between the HRAS mutation and the habits was statistically significant (p = 0.002) [25].

**Table 3:** Characteristics of the included studies This table shows the included studies, study design, sample size, study population, statistical analysis, and parameters used.

S. No	Author	Year	Study design	Sample size	Study population	Sample used	Parameters used	Statistical analysis
1.	Chang et al. [17]	2014	Cross-sectional	79	Taiwanese	Tissue	Multiplex PCR, primer extension analysis	Two-sided Fisher’s exact test
2.	Murugan et al. [18]	2009	Cross-sectional	56	Vietnamese	Tissue	Polymerase chain reaction	Fisher’s exact test
3.	Warnakulasuriya et al. [19]	1992	Cross-sectional	48	Caucasoid	Tissue	PCR, oligonucleotide probe hybridization	-
4.	Zanaruddin et al. [20]	2013	Cross-sectional	123	Asian	Tissue and cell line	PCR, direct sequencing	Kaplan-Meier survival analysis
5.	Saranath et al. [21]	1991	Cross-sectional	57	Indian	Tissue	PCR, oligonucleotide probe hybridization	-
6.	Munirajan et al. [22]	1998	Cross-sectional	46	Indian	Tissue	SSCP analysis, direct DNA sequencing	-
7.	Sathyan et al. [23]	2007	Cross-sectional	152	Arabian	Tissue	PCR-SSCP, direct DNA sequencing, IHC	Chi-square test, log-rank test, Kaplan-Meier method
8.	Koumaki et al. [24]	2012	Case-control study	166; Cases: 86; controls: 80	Greek	Tissue	Nested PCR, direct sequencing	Chi-square test
9.	Uchibori et al. [25]	2021	Case-control study	168; Cases: 84; controls: 84	Japanese	Tissue	PCR, Sanger sequencing	Chi-square test

Discussion

Oral carcinogenesis is a complex phenomenon in which altered genetic events lead to the disruption of normal regulatory pathways. The malignant transformation of the epithelial cells is due to the dysregulation in the biochemical and molecular events [26]. The cell biology of the oral epithelial cells is controlled by the signal transduction pathways. These signaling pathways are mainly governed by the oncogenes. When these oncogenes get mutated, it leads to overproduction of the excitatory proteins altering these signaling pathways that lead to disturbance in cellular proliferation and differentiation, causing cell death [27]. The first step in oncogenesis is the process of phosphorylation of proteins using the amino acids serine, threonine, and tyrosine as substrates. The second process involves GTPases delivering the signals, while the third involves DNA controlling transcription [28]. One of the oncogenes with the most frequent mutations across all malignancies is RAS. HRAS is one of the members of the RAS superfamily.

Numerous studies have confirmed the relationship between mutated HRAS and OSCC. A recent study reported computational landscapes of the HRAS mutation from four data sets namely the MD Anderson Cancer Center cohort (MDACC), Kura cohort (KURA KO-TIP-001 trial participants. ClinicalTrials.gov identifier: NCT02383927), Foundation Medicine cohort (FMI-profiled cases [samples collected from 2013 to 2020]), and AACR GENIE v.12 cohort (AACR Project GENIE v.12,10 an international clinico-genomic data-sharing consortium). It showed that there were alterations in codons 12 and 13 in 59% of the HRAS mutation in the MDACC dataset, 70% in the KURA dataset, and 68% in the FMI dataset. In the FMI dataset, codon 13 also included two frameshift mutations showing G13_V14. It was also found that in all cohorts, the most common mutation encountered was G12S, which was 25% in MDACC, 30% in Kura, 26% in the FMI data set, and 41% in AACR Project GENIE v.12 cohort, respectively. Other than codon 12 and codon 13, codon 61 was also frequently reported for mutation which was presented as 31% in the MDACC, 11% in Kura, 27% in the FMI data set, and 20% in AACR Project GENIE v.12 cohort [29].

HRAS mutation, which included two different point mutations guanine to adenine (G to A) and guanine to thymine (G to T) in codon 12, was seen in OSCC cases [30]. Tadokoro et al. in 1989 found a mutation in codon 13 guanine to cytosine (G to C) of the HRAS gene in an OSCC cell line [31]. A study demonstrated glycine to thymine (G to T) transversion, glycine to adenine (G to A) transition, and glycine to cytosine (G to C) transversion in OSCC [32]. The steps behind the involvement of HRAS mutation in the pathogenesis of OSCC are under research.

Prolonged binding of the ATP to RAS makes the RAS in its active form leading to mutation of RAS. Three crucial steps involved in membrane localization and activation of RAS are farnesylation, palmitoylation, and ubiquitination. Farnesylation is one of the most important steps in the post-translational modifications of the proteins [33]. It is the process in which the isoprenoid farnesyl phosphate is attached to the target proteins during which a 15-carbon farnesyl lipid group is transferred onto one or more cysteine residues. The farnesylated proteins play a key role in the cell division cycle, migration, cytoskeleton organization, survival, embryogenesis, and adult homeostasis which was established in a study done on animal models in 2005 [34]. Palmitoylation is the process of attachment of the 16-carbon palmitate covalently attaching to cysteine residues throughout the protein via thioether bonds. It plays an important role in reversibly altering the localization, stability, and function of numerous proteins [35]. It is noted that HRAS is the highest irreversibly palmitoylated protein [36]. When the sequence of palmitoylation gets affected, it alters the localization and stability of the proteins. Ubiquitination is the process of attachment of ubiquitin molecules to the protein substrates. It is very important for the functional activity of the proteins, DNA damage repair, and cell-cycle progression [37]. Impaired ubiquitination leads to dysregulation of the metabolic reprogramming of the cells which leads to increased demand in meeting up the energy required for normal cellular processes [38].

The results of the included studies in our systematic review showed around 9.6% of novel HRAS gene mutations among the 895 participants enrolled. These activated HRAS mutations have high oncogenic potential to activate the RAS MAPK signaling pathway through phosphorylation by interactions with small GTP binding proteins in response to the external stimulus. It has the potent ability to accumulate higher levels of GTP making the HRAS gene remain in its activated state. The transcription factors that are effectors of cellular responses to MAPK pathway activation are among the numerous substrates that are activated when the MAPK signaling pathway is activated through phosphorylation [39]. Both positive and negative feedback mechanisms control the MAPK pathway. By amplifying the stimulus, positive feedback raises the sensitivity of the system to the signaling inputs [40]. The growth factor receptor binding protein 2 (GRB2) and the GRB2-associated binding protein 1 (GAB1) also work to activate the src homology 2 (SH2) domain-containing protein tyrosine phosphatase 2 (SHP2) phosphatase, which aids in the dephosphorylation of Ras GTPase activating protein (RasGAP) docking sites on GAB1, which in turn decreases RAS inactivation and increases MAPK signaling [41]. This occurs at the level of GAB protein 1, which is recruited at the activation of RTKs. The negative regulators also regulate the MAPK pathway by reducing the time that they are active. The MAPK phosphatases, the sporty proteins, and scaffolding proteins like 14-3-3, which control the RAF's cellular localization and stability, all act as inhibitory regulators to balance this pathway. Additionally, the SOS proteins are phosphorylated by ERKs, which impair the SOS-GRB2 connections and reduce the recruitment of the SOS to the membrane, which inhibits the activation of RAS. After that, the ERK phosphorylates the rapidly accelerated fibrosarcoma (RAF), which results in lower RAF kinase activity and decreased MEK and ERK activation [42].

Cellular outcomes of HRAS mutation

The main mechanism of the ERK1/2 signaling pathway for influencing tumor cells is by controlling cell proliferation. Persistent ERK activation can cause pro-differentiation signals and cell-cycle suppression in cells of epithelial origin [43]. The crucial cell-cycle regulatory event is the transition from G0 to G1/S. Sustained activation and nuclear localization of ERK1/2 may have an impact on cyclin D1 synthesis. A study investigated the association of cell-cycle regulatory proteins and OSCC, which proved that there is a significant association between cell-cycle regulators like cyclin D1, CDK4, Rb, and p16 with OSCC [22]. Either an increased positive feedback mechanism or impaired negative regulators might have made the downstream members of the MAPK signaling pathway less active. Impaired negative regulators might have affected the inhibitory regulators because of which the SOS recruitment to the membrane may have remained high, leading to the continued activation of RAS and impaired farnesylation, palmitoylation, and ubiquitination making the signaling pathway maintain continuous positive feedback. This might have led to increased cellular proliferation and differentiation ending in carcinogenesis.

It was also seen that the Taiwanese population showed a combination of HRAS and PIK3CA mutation. This is because the MAPK pathway and the PI3K signaling pathways interact to promote the growth and survival of transformed cells. Increased PI3K signaling is mainly due to RTK activation, causing amplification of the genes encoding the key components of the signaling pathway. PIK3CA is one of the most commonly mutated genes of the PI3K signaling pathway. The MAPK pathway regulates the mammalian target of rapamycin (mTOR) through the inactivation of tuberous sclerosis complex 1 (TSC1)/tuberous sclerosis complex 2 (TSC2). This process is regulated with the help of ERK [44]. The phosphorylation of ERK leads to the dissociation of TSC1/TSC2, which in turn impairs the ability of TSC2 to inhibit the mTOR signaling. This also activates the regulatory associated protein of mTOR (RPTOR), an essential scaffolding protein of the mTORC1 complex after which the MAPK and the PI3K signaling pathways converge on the B-cell leukemia 2 homology domain 3 (BH3) family of proteins, which plays an important role in apoptosis [45]. Though this systematic review has been done comprehensively, it has its limitations.

Limitations

The included studies have been done on a diverse population. Studies conducted in the same population will give a better understanding of the disease state. Future research needs to be conducted within the same population to get better results.

## Conclusions

To summarize, this systematic review has given an extensive idea about the effect of HRAS mutation in OSCC. The mutation or the activation of HRAS for a longer period leads to oncogenesis. Though numerous studies have been conducted on the carcinogenic effect of HRAS mutation on OSCC that have given us an elaborate idea about the involvement of the HRAS mutation in the pathogenesis of OSCC, doubts about the role of HRAS mutation and the process of farnesylation, palmitoylation, and ubiquitination in the pathogenesis of OSCC are still left unexplored. Understanding the important steps of farnesylation, palmitoylation, and ubiquitination will enable future studies to address the discrepancies. Further, it can be understood that the proteins of the RAS pathway can be used as prognostic markers in other cancers but research must be conducted in OSCC to investigate the molecular role and pathogenesis of the RAS pathway. This will in turn enable future researchers to find a targeted therapy for RAS-mediated OSCC.
